# (*E*)-*N*′-(3,5-Dichloro-2-hy­droxy­benzyl­idene)-2-meth­oxy­benzohydrazide

**DOI:** 10.1107/S160053681102366X

**Published:** 2011-06-25

**Authors:** Xiao-Yan Li

**Affiliations:** a Zibo Vocational Institute, Zibo 255314, People’s Republic of China

## Abstract

In the title compound, C_15_H_12_Cl_2_N_2_O_3_, the dihedral angle between the two substituted aromatic rings is 5.4 (4)°. Intra­molecular O—H⋯N and N—H⋯O hydrogen bonds affect the planarity of the molcular conformation, with a mean deviation from the plane defined by the non-H atoms of 0.062 (2) Å. The mol­ecule exists in a *trans* configuration with respect to the methyl­idene unit. In the crystal, mol­ecules are linked by N—H⋯O inter­actions.

## Related literature

For the crystal structures of hydrazone compounds, see: Li (2011 ▶); Hashemian *et al.* (2011 ▶); Lei (2011 ▶); Shalash *et al.* (2010 ▶).
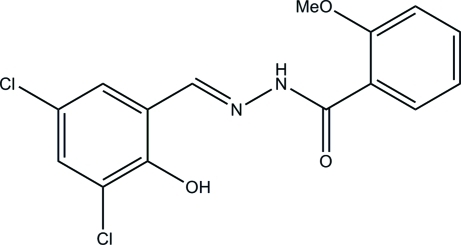


## Experimental

### 

#### Crystal data

C_15_H_12_Cl_2_N_2_O_3_
*M* *_r_* = 339.17
Monoclinic, 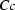

*a* = 10.845 (7) Å
*b* = 12.771 (8) Å
*c* = 10.856 (7) Å
β = 96.683 (7)°
*V* = 1493.4 (16) Å^3^
*Z* = 4
Mo *K*α radiation
μ = 0.45 mm^−1^
*T* = 298 K
0.18 × 0.18 × 0.17 mm

#### Data collection

Bruker SMART CCD area-detector diffractometer
Absorption correction: multi-scan (*SADABS*; Sheldrick, 1996 ▶) *T*_min_ = 0.924, *T*_max_ = 0.928
4586 measured reflections
2978 independent reflections
2011 reflections with *I* > 2σ(*I*)
*R*_int_ = 0.050

#### Refinement

*R*[*F* ^2^ > 2σ(*F* ^2^)] = 0.058
*wR*(*F* ^2^) = 0.133
*S* = 1.02
2978 reflections
204 parameters
4 restraints
H atoms treated by a mixture of independent and constrained refinement
Δρ_max_ = 0.28 e Å^−3^
Δρ_min_ = −0.28 e Å^−3^
Absolute structure: Flack (1983 ▶), 1272 Friedel pairs
Flack parameter: 0.10 (10)

### 

Data collection: *SMART* (Bruker, 1998 ▶); cell refinement: *SAINT* (Bruker, 1998 ▶); data reduction: *SAINT*; program(s) used to solve structure: *SHELXS97* (Sheldrick, 2008 ▶); program(s) used to refine structure: *SHELXL97* (Sheldrick, 2008 ▶); molecular graphics: *SHELXTL* (Sheldrick, 2008 ▶); software used to prepare material for publication: *SHELXL97*.

## Supplementary Material

Crystal structure: contains datablock(s) global, I. DOI: 10.1107/S160053681102366X/om2438sup1.cif

Structure factors: contains datablock(s) I. DOI: 10.1107/S160053681102366X/om2438Isup2.hkl

Supplementary material file. DOI: 10.1107/S160053681102366X/om2438Isup3.cml

Additional supplementary materials:  crystallographic information; 3D view; checkCIF report

## Figures and Tables

**Table 1:** Hydrogen-bond geometry (Å, °)

*D*—H⋯*A*	*D*—H	H⋯*A*	*D*⋯*A*	*D*—H⋯*A*
O1—H1⋯N1	0.82	1.82	2.543 (4)	146
N2—H2⋯O3	0.90 (1)	2.02 (5)	2.624 (4)	123 (5)
N2—H2⋯O1^i^	0.90 (1)	2.63 (4)	3.271 (5)	129 (5)

Symmetry code: (i) 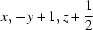.

**Fig. 1.**
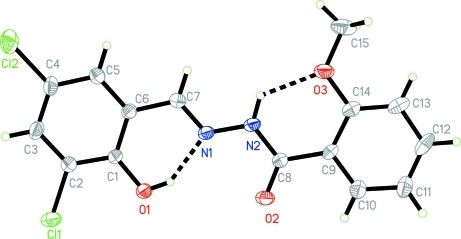
The molecular structure with displacement parameters drawn at the 30% probability level. Hydrogen bonds are indicated by dashed lines.

## supplementary crystallographic information

### Comment

In the last few years, hydrazones have been attracted much attention for their crystal structures (Li, 2011; Hashemian *et al.*, 2011; Lei, 2011; Shalash *et al.*, 2010).

In the crystal structure of the title hydrazone molecule, as shown in Fig. 1, the dihedral angle between the two substituted aromatic rings is 5.4 (4)°. The intramolecular O—H···N and N—H···O hydrogen bonds (Table 1) affect the planarity of the conformation of the molecule. The molecule exists in a *trans* configuration with respect to the methylidene unit.

### Experimental

A mixture of 2-methoxybenzhydrazide (0.166 g, 1 mmol) and 3,5-dichlorosalicylaldehyde (0.190 g, 1 mmol) in 30 ml of ethanol containing a few drops of acetic acid was refluxed for about 1 h. On cooling to room temperature, a solid precipitate was formed. The solid was filtered and then recrystallized from methanol. Colorless crystals suitable for X-ray diffraction were obtained by slow evaporation of the solution.

### Refinement

The and N-bound hydrogen atom was located from a difference Fourier map and refined isotropically. The rest of hydrogen atoms were positioned geometrically [C—H = 0.93 & 0.96 Å; O—H = 0.82 Å] and refined using a riding model [U_iso_(H) = 1.2U_eq_(C) and 1.5 U_eq_(C15 and O1)]. A rotating-group model was applied for the methyl group.

### Crystal data

**Table:**

C_15_H_12_Cl_2_N_2_O_3_	*F*(000) = 696
*M**_r_* = 339.17	*D*_x_ = 1.508 Mg m^−^^3^
Monoclinic, *C**c*	Mo *K*α radiation, λ = 0.71073 Å
Hall symbol: C -2yc	Cell parameters from 816 reflections
*a* = 10.845 (7) Å	θ = 2.4–24.3°
*b* = 12.771 (8) Å	µ = 0.45 mm^−^^1^
*c* = 10.856 (7) Å	*T* = 298 K
β = 96.683 (7)°	Block, colorless
*V* = 1493.4 (16) Å^3^	0.18 × 0.18 × 0.17 mm
*Z* = 4	

### Data collection

**Table:**

Bruker SMART CCD area-detector diffractometer	2978 independent reflections
Radiation source: fine-focus sealed tube	2011 reflections with *I* > 2σ(*I*)
graphite	*R*_int_ = 0.050
ω scans	θ_max_ = 27.5°, θ_min_ = 2.5°
Absorption correction: multi-scan (*SADABS*; Sheldrick, 1996)	*h* = −14→13
*T*_min_ = 0.924, *T*_max_ = 0.928	*k* = −16→12
4586 measured reflections	*l* = −13→14

### Refinement

**Table:**

Refinement on *F*^2^	Secondary atom site location: difference Fourier map
Least-squares matrix: full	Hydrogen site location: inferred from neighbouring sites
*R*[*F*^2^ > 2σ(*F*^2^)] = 0.058	H atoms treated by a mixture of independent and constrained refinement
*wR*(*F*^2^) = 0.133	*w* = 1/[σ^2^(*F*_o_^2^) + (0.0425*P*)^2^] where *P* = (*F*_o_^2^ + 2*F*_c_^2^)/3
*S* = 1.02	(Δ/σ)_max_ < 0.001
2978 reflections	Δρ_max_ = 0.28 e Å^−^^3^
204 parameters	Δρ_min_ = −0.28 e Å^−^^3^
4 restraints	Absolute structure: Flack (1983), 1272 Friedel pairs
Primary atom site location: structure-invariant direct methods	Flack parameter: 0.10 (10)

### Special details

**Table:**

Geometry. All esds (except the esd in the dihedral angle between two l.s. planes) are estimated using the full covariance matrix. The cell esds are taken into account individually in the estimation of esds in distances, angles and torsion angles; correlations between esds in cell parameters are only used when they are defined by crystal symmetry. An approximate (isotropic) treatment of cell esds is used for estimating esds involving l.s. planes.
Refinement. Refinement of F^2^ against ALL reflections. The weighted R-factor wR and goodness of fit S are based on F^2^, conventional R-factors R are based on F, with F set to zero for negative F^2^. The threshold expression of F^2^ > 2sigma(F^2^) is used only for calculating R-factors(gt) etc. and is not relevant to the choice of reflections for refinement. R-factors based on F^2^ are statistically about twice as large as those based on F, and R- factors based on ALL data will be even larger.

### Fractional atomic coordinates and isotropic or equivalent isotropic displacement parameters (Å^2^)

**Table:**

	*x*	*y*	*z*	*U*_iso_*/*U*_eq_	
Cl1	0.40120 (15)	0.77322 (10)	0.22803 (13)	0.0652 (4)	
Cl2	0.47525 (14)	0.96477 (10)	0.67126 (14)	0.0658 (4)	
N1	0.6075 (4)	0.4977 (3)	0.5532 (3)	0.0389 (9)	
N2	0.6498 (4)	0.4042 (3)	0.6052 (3)	0.0436 (10)	
O1	0.5144 (3)	0.5940 (2)	0.3592 (3)	0.0461 (8)	
H1	0.5450	0.5443	0.3998	0.069*	
O2	0.6238 (4)	0.3303 (2)	0.4167 (3)	0.0581 (10)	
O3	0.7634 (3)	0.2894 (3)	0.7842 (3)	0.0535 (10)	
C1	0.5091 (4)	0.6771 (3)	0.4333 (4)	0.0353 (10)	
C2	0.4584 (4)	0.7706 (4)	0.3839 (4)	0.0436 (12)	
C3	0.4487 (4)	0.8590 (4)	0.4551 (5)	0.0464 (12)	
H3	0.4142	0.9202	0.4198	0.056*	
C4	0.4913 (5)	0.8547 (4)	0.5798 (4)	0.0450 (12)	
C5	0.5424 (4)	0.7637 (4)	0.6342 (4)	0.0418 (12)	
H5	0.5708	0.7621	0.7184	0.050*	
C6	0.5504 (4)	0.6748 (3)	0.5611 (4)	0.0360 (10)	
C7	0.6003 (4)	0.5782 (4)	0.6203 (4)	0.0421 (11)	
H7	0.6261	0.5760	0.7050	0.051*	
C8	0.6559 (4)	0.3220 (3)	0.5280 (4)	0.0376 (11)	
C9	0.7044 (4)	0.2206 (3)	0.5831 (4)	0.0374 (11)	
C10	0.6993 (5)	0.1355 (4)	0.5042 (5)	0.0476 (12)	
H10	0.6651	0.1447	0.4223	0.057*	
C11	0.7425 (5)	0.0378 (4)	0.5408 (5)	0.0595 (15)	
H11	0.7356	−0.0183	0.4859	0.071*	
C12	0.7965 (5)	0.0254 (4)	0.6618 (6)	0.0646 (16)	
H12	0.8266	−0.0400	0.6882	0.077*	
C13	0.8063 (5)	0.1080 (4)	0.7436 (5)	0.0548 (14)	
H13	0.8445	0.0990	0.8242	0.066*	
C14	0.7587 (4)	0.2053 (4)	0.7049 (4)	0.0427 (12)	
C15	0.8242 (5)	0.2795 (5)	0.9067 (5)	0.0705 (18)	
H15A	0.7862	0.2242	0.9488	0.106*	
H15B	0.8173	0.3441	0.9505	0.106*	
H15C	0.9103	0.2634	0.9035	0.106*	
H2	0.667 (5)	0.404 (5)	0.6884 (9)	0.085*	

### Atomic displacement parameters (Å^2^)

**Table:**

	*U*^11^	*U*^22^	*U*^33^	*U*^12^	*U*^13^	*U*^23^
Cl1	0.0949 (10)	0.0518 (8)	0.0441 (7)	0.0028 (8)	−0.0130 (6)	0.0124 (6)
Cl2	0.0854 (10)	0.0383 (6)	0.0762 (10)	−0.0036 (7)	0.0194 (7)	−0.0199 (7)
N1	0.053 (2)	0.033 (2)	0.0301 (19)	0.0056 (18)	−0.0001 (17)	0.0040 (16)
N2	0.059 (3)	0.035 (2)	0.034 (2)	0.0087 (19)	−0.005 (2)	0.0070 (18)
O1	0.074 (2)	0.0319 (18)	0.0310 (17)	0.0013 (16)	−0.0010 (16)	−0.0017 (14)
O2	0.086 (3)	0.048 (2)	0.0353 (19)	0.0129 (19)	−0.0132 (18)	0.0007 (17)
O3	0.066 (2)	0.054 (2)	0.038 (2)	0.0143 (18)	−0.0076 (17)	0.0048 (17)
C1	0.041 (3)	0.033 (2)	0.032 (2)	−0.0051 (19)	0.005 (2)	0.0042 (19)
C2	0.055 (3)	0.038 (3)	0.037 (3)	−0.006 (2)	0.001 (2)	0.001 (2)
C3	0.052 (3)	0.030 (3)	0.057 (3)	−0.003 (2)	0.005 (2)	0.004 (2)
C4	0.054 (3)	0.032 (3)	0.051 (3)	−0.008 (2)	0.014 (3)	−0.007 (2)
C5	0.049 (3)	0.039 (3)	0.037 (3)	−0.002 (2)	0.003 (2)	−0.004 (2)
C6	0.042 (3)	0.032 (2)	0.034 (3)	0.000 (2)	0.006 (2)	0.0013 (19)
C7	0.051 (3)	0.043 (3)	0.032 (2)	−0.003 (2)	0.002 (2)	0.001 (2)
C8	0.040 (3)	0.035 (2)	0.037 (3)	0.002 (2)	0.000 (2)	0.003 (2)
C9	0.041 (3)	0.030 (3)	0.041 (3)	0.0035 (19)	0.007 (2)	0.0096 (19)
C10	0.053 (3)	0.040 (3)	0.051 (3)	−0.003 (2)	0.011 (2)	−0.001 (2)
C11	0.072 (4)	0.035 (3)	0.072 (4)	0.001 (2)	0.011 (3)	−0.003 (3)
C12	0.063 (4)	0.038 (3)	0.094 (5)	0.017 (3)	0.015 (3)	0.022 (3)
C13	0.056 (3)	0.055 (3)	0.054 (3)	0.010 (3)	0.008 (3)	0.024 (3)
C14	0.043 (3)	0.042 (3)	0.043 (3)	0.007 (2)	0.007 (2)	0.011 (2)
C15	0.070 (4)	0.096 (5)	0.042 (3)	0.020 (3)	−0.010 (3)	0.007 (3)

### Geometric parameters (Å, °)

**Table:**

Cl1—C2	1.734 (5)	C5—H5	0.9300
Cl2—C4	1.741 (5)	C6—C7	1.465 (6)
N1—C7	1.268 (5)	C7—H7	0.9300
N1—N2	1.377 (5)	C8—C9	1.496 (6)
N2—C8	1.350 (5)	C9—C10	1.381 (6)
N2—H2	0.900 (7)	C9—C14	1.397 (6)
O1—C1	1.337 (5)	C10—C11	1.375 (7)
O1—H1	0.8200	C10—H10	0.9300
O2—C8	1.223 (5)	C11—C12	1.383 (8)
O3—C14	1.373 (6)	C11—H11	0.9300
O3—C15	1.420 (6)	C12—C13	1.376 (8)
C1—C2	1.396 (6)	C12—H12	0.9300
C1—C6	1.407 (5)	C13—C14	1.392 (6)
C2—C3	1.379 (7)	C13—H13	0.9300
C3—C4	1.380 (6)	C15—H15A	0.9600
C3—H3	0.9300	C15—H15B	0.9600
C4—C5	1.390 (6)	C15—H15C	0.9600
C5—C6	1.394 (6)		
			
C7—N1—N2	120.5 (4)	O2—C8—N2	121.2 (4)
C8—N2—N1	117.2 (3)	O2—C8—C9	121.1 (4)
C8—N2—H2	127 (4)	N2—C8—C9	117.7 (4)
N1—N2—H2	116 (4)	C10—C9—C14	117.3 (4)
C1—O1—H1	109.5	C10—C9—C8	116.5 (4)
C14—O3—C15	119.8 (4)	C14—C9—C8	126.1 (4)
O1—C1—C2	119.4 (4)	C11—C10—C9	123.1 (5)
O1—C1—C6	123.0 (4)	C11—C10—H10	118.4
C2—C1—C6	117.6 (4)	C9—C10—H10	118.4
C3—C2—C1	122.5 (4)	C10—C11—C12	118.1 (5)
C3—C2—Cl1	119.3 (4)	C10—C11—H11	121.0
C1—C2—Cl1	118.2 (4)	C12—C11—H11	121.0
C2—C3—C4	118.6 (4)	C13—C12—C11	121.2 (5)
C2—C3—H3	120.7	C13—C12—H12	119.4
C4—C3—H3	120.7	C11—C12—H12	119.4
C3—C4—C5	121.4 (4)	C12—C13—C14	119.4 (5)
C3—C4—Cl2	118.9 (4)	C12—C13—H13	120.3
C5—C4—Cl2	119.6 (4)	C14—C13—H13	120.3
C4—C5—C6	119.2 (4)	O3—C14—C13	121.5 (4)
C4—C5—H5	120.4	O3—C14—C9	117.7 (4)
C6—C5—H5	120.4	C13—C14—C9	120.8 (5)
C5—C6—C1	120.7 (4)	O3—C15—H15A	109.5
C5—C6—C7	118.7 (4)	O3—C15—H15B	109.5
C1—C6—C7	120.6 (4)	H15A—C15—H15B	109.5
N1—C7—C6	118.3 (4)	O3—C15—H15C	109.5
N1—C7—H7	120.8	H15A—C15—H15C	109.5
C6—C7—H7	120.8	H15B—C15—H15C	109.5

### Hydrogen-bond geometry (Å, °)

**Table:**

*D*—H···*A*	*D*—H	H···*A*	*D*···*A*	*D*—H···*A*
O1—H1···N1	0.82	1.82	2.543 (4)	146
N2—H2···O3	0.90 (1)	2.02 (5)	2.624 (4)	123 (5)
N2—H2···O1^i^	0.90 (1)	2.63 (4)	3.271 (5)	129 (5)

Symmetry codes: (i) *x*, −*y*+1, *z*+1/2.
